# Stability of unfrozen whole blood DNA for remote genotypic analysis of HIV-1 coreceptor tropism

**DOI:** 10.1186/1471-2334-13-508

**Published:** 2013-10-30

**Authors:** Genny Meini, Angelo Materazzi, Francesco Saladini, Andrea Rosi, Ilaria Vicenti, Michele Mancini, Antonella Pirazzoli, Cinzia Caudai, Maurizio Zazzi

**Affiliations:** 1 Department of Medical Biotechnologies, University of Siena, Siena, Italy; 2 ViiV Healthcare, Verona, Italy

**Keywords:** HIV-1, V3 region, Coreceptor tropism, DNA, Whole blood, Stability, Storage, Shipment

## Abstract

**Background:**

Maraviroc is an HIV-1 coreceptor antagonist that has shown good efficacy and tolerability in treatment-naive and treatment-experienced patients harboring CCR5-tropic virus. The use of Maraviroc in treatment simplification in patients with suppressed plasma HIV-1 RNA requires analysis of HIV-1 DNA. Coreceptor tropism testing is often performed remotely at reference laboratories. In this study paired whole blood stored at + 4°C and at−20°C were compared as a source for genotypic coreceptor tropism testing.

**Methods:**

Two hundred paired whole blood samples from different patients were analysed. Each sample was stored in two different conditions: one aliquot was stored at−20°C until spin column DNA extraction (WB20) and one aliquot was stored at +4°C for two weeks and then placed at room temperature (22-24°C) for two days before DNA extraction (WB4). Subsequently, a fragment encompassing the HIV-1 gp120 V3 domain was amplified by a singlicate nested PCR followed by triplicate nested PCR in the negative samples. A randomly selected panel of 20 paired WB4 and WB20 duplicate amplification products were sequenced and coreceptor tropism was inferred by geno2pheno [coreceptor].

**Results:**

WB20 yielded a higher amount of DNA than WB4 (median [IQR] values 332.5 ng/μl [117.5-401] and 107 ng/μl [56.6-318], respectively; P < 0.001). However, the DNA purity was higher for WB4 than for WB20 (median distance from the optimal OD_260/280_ ratio, 0.14 [0.07-0.79] and 0.96 [0.36-1.10], respectively; P < 0.0001). The number of samples successfully amplified was 152 (76.0%) for WB20 and 155 (77.5%) for WB4 with the first PCR and 179 (89.5%) for WB20 and 181 (90.5%) for WB4 (P = ns) following subsequent triplicate analysis. The inferred coreceptor tropism was concordant in 18 out of 20 paired WB4 and WB20 samples. Two samples yielded discordant results, consistent with the discordance rate within duplicates from the same sample source (2/20 with WB4 and 1/20 with WB20) due to the inherent gp120 V3 variability.

**Conclusions:**

Storing whole blood at +4°C for up to two weeks and shipping at room temperature is a convenient method for obtaining HIV-1 gp120 V3 sequence information via testing at a remote laboratory in patients with suppressed viremia.

## Background

Cell infection by the Human Immunodeficiency Virus type 1 (HIV-1) requires interaction between the viral envelope surface glycoprotein (gp120) and a cellular coreceptor (CCR5 or/and CXCR4) together with the CD4 receptor molecule. HIV-1 can use CCR5 (R5 virus), or CXCR4 (×4 virus) or both (dual mixed or DM virus) coreceptors to enter target cells [1]. Determination of HIV-1 coreceptor usage is required prior to therapy with Maraviroc, the only CCR5 antagonist currently licensed for treatment of HIV-1 infection [2]. Presently, genotypic tropism testing is the most widely used method in Europe to define HIV-1 coreceptor tropism, based on the strict correlation between HIV-1 V3 *env* region sequence and coreceptor usage [3].

In Europe, Maraviroc is licensed for therapy-experienced patients but not yet for first-line therapy. Maraviroc containing regimens are also used in patients with suppressed viremia [4]. This strategy is supported by the safety profile of this drug, decreasing treatment toxicity [5]. In such patients, however, HIV coreceptor tropism cannot be determined on plasma RNA as recommended but proviral DNA can be considered as an alternative source of viral genetic material [6]. Previous studies have indeed shown a good correlation between genotype based tropism results obtained from paired HIV-1 DNA and RNA [7,8] and preliminary evidence of the clinical relevance of proviral HIV-1 DNA tropism testing in the context of suppressed viremia has been provided [9,10].

While genotypic coreceptor tropism testing is gaining wide acceptance, this procedure may not always be available in all clinical settings. Standard sample handling for remote testing requires storage of frozen specimens and shipment in dry ice, adding complexity to routine analysis. In this study, whole blood storage at +4°C and shipment at room temperature was evaluated as a more convenient handling method for remote HIV-1 DNA coreceptor tropism testing. To test this strategy, 200 paired whole blood samples were analysed.

## Methods

A total of 200 whole blood samples were collected from 200 patients with suppressed viremia as defined as HIV-1 RNA <40 copies/ml by the Abbott RealTime assay. Patients signed an informed consent allowing anonymous use of samples for research purposes and the study was approved by the Ethical Committee of the Siena University Hospital. Of these, 43 had HIV-1 RNA target detected and 157 had HIV-1 RNA target not detected. For each sample, (i) one 500-microliter whole blood aliquot was frozen within 4 hours after drawing and stored at−20°C until DNA extraction (WB20) and (ii) one 500-microliter whole blood aliquot was stored at +4°C for two weeks within 4 hours after drawing, then placed at room temperature (22-24°C) for two days (WB4) and subjected to the same DNA extraction procedure.

Whole blood DNA was extracted by using the High Pure Viral Nucleic Acid Kit (Roche Applied Science, catalogue number 11858874001) following the manufacturer instructions. The choice of this system was based on previous comparisons showing that DNA yield is increased with respect to the QIAamp DNA Blood Mini Kit (Qiagen) (data not shown). To make the procedure as straightforward as possible, the DNA extracted was not measured and 5 of the total 50 microliters obtained from the extraction procedure were directly used for PCR. However, DNA concentration and purity were subsequently measured spectrophotometrically (NanoPhotometer P360, Implen) as a post hoc analysis to compare the yield of the two extraction procedures.

A 421-bp fragment encompassing the HIV-1 gp120 V3 domain was amplified by nested PCR. Blood DNA extracted from WB4 or WB20 was amplified by a nested PCR protocol using primer P150 (5′-AATGTCAGCACAGTACAATGYACACAT-3′, coordinates 6945-6971 in the reference HXB2 genome) and P151 (5′-CTACTTTATATTTATATAATTCAYTTCTC-3′, 7661-7689) in the outer amplification step and primer P537 (5′-CAGTACARTGYACACATGGAAT-3′, 6955-6976) and P538 (5′-TAGAAAAATTCYCCTCYACAATTAAA-3′, 7350-7375) in the inner amplification step. Both outer and inner PCR mixtures contained 50 mM Tris–HCl (pH 9.0 at 25°C), 50 mM KCl, 2 mM MgCl_2_, 200 μM each dNTP, 1.25 U GoTaq polymerase (Promega) and 8 pmol each primer. After an initial denaturation step of 3 minutes at 94°C, the cycling profile was 20 seconds at 52°C, 40 seconds at 72°C and 30 seconds at 94°C for both steps but the number of cycles was 25 in the outer PCR and 30 in the inner PCR. PCR products were resolved on 1.5% agarose gels and stained with ethidium bromide.

Each DNA extract was first subjected to a singlicate amplification. When the expected PCR product was not obtained, a triplicate amplification was performed again using 5 μl as the template, to increase overall sensitivity of the assay.

To ensure that the differences in the two sample processing procedures do not have any impact on the distribution of the virus variants with different coreceptor tropism, a panel of 20 paired WB4 and WB20 amplification products were randomly selected and subjected to bidirectional Sanger sequencing using the same inner PCR primers P537 and P538 and BigDye 1.1 chemistry. Sequencing products were resolved on an ABI3130xl instrument (Applied Biosystems, Foster, CA, USA) and handled by the SeqMan module of the DNASTAR 7.1.0 package (DNASTAR Inc., Madison, WI, USA). Since intrasample variability in the V3 region using population sequencing has been reported [11], two distinct PCR products were analyzed for each of the cases selected for the WB4 vs. WB20 comparison. Coreceptor tropism was assigned by the geno2pheno [coreceptor] algorithm with a 10% False Positive Rate cut-off [12], based on the lowest of the two FPR values obtained for each sample.

## Results and discussion

The number of samples successfully amplified by the complete procedure was 179 (89.5%) for WB20 and 181 (90.5%) for WB4 (P = ns; chi-square test) (Table 1). There was no significant difference in the rate of positive samples with WB4 and WB20, either in the first singlicate test or in the subsequent triplicate analysis performed on negative samples. Table 2 shows the details of the first singlicate amplification and the second triplicate amplification on the 200 paired WB4 and WB20 samples. The rate of positive reactions in the subset of samples tested by triplicate amplification was 48.9% (66/135 reactions) for WB4 and 38.9% (56/144) for WB20 (P = 0.116; chi-square test).

**Table 1:** Amplification of the gp120 V3 sequence from the 200 whole blood samples stored at +4°C (WB4) and at −20°C (WB20) using a first singlicate plus second triplicate testing approach

**Sample type**	**Positive with first singlicate test**	**Positive with second triplicate test**	**Negative with both first singlicate and second triplicate test**	**Overall % of positive cases**
WB4	155	26	19	90.5
WB20	152	27	21	89.5

**Table 2:** Distribution of results with the first singlicate and the second triplicate testing on the 200 whole blood samples stored at +4°C (WB4) and at−20°C (WB20)

**First singlicate test**		**Second triplicate test**		**No. of cases**	**% of cases**
**WB4**	**WB20**	**WB4**	**WB20**		
Positive	Positive	NA	NA	133	66.5
Positive	Negative	NA	Positive	19	9.5
Positive	Negative	NA	Negative	3	1.5
Negative	Positive	Positive	NA	16	8.0
Negative	Positive	Negative	NA	3	1.5
Negative	Negative	Positive	Positive	7	3.5
Negative	Negative	Positive	Negative	3	1.5
Negative	Negative	Negative	Positive	1	0.5
Negative	Negative	Negative	Negative	15	7.5

NA, not applicable (second triplicate test run only on samples which were negative in first singlicate test).

Measurement of all the DNA samples by spectrophotometry detected two WB4 cases and six WB20 cases where the DNA concentration fell below the threshold of sensitivity of the system. However, in each case the corresponding WB20 and WB4 sample, respectively, had detectable DNA. Paired analysis of DNA concentration for the 192 cases with measurable DNA in both WB4 and WB20 showed that WB20 yielded a higher amount of DNA compared to WB4 (median [IQR] values 332 ng/μl [117-401] and 107 ng/μl [57-318]; P < 0.001, Wilcoxon signed rank test). By contrast, the DNA purity (as measured by absolute distance from the optimal 1.8-2.0 OD_260/280_ ratio [13]) was higher for WB4 than for WB20 (median distance 0.14 [0.07-0.79] and 0.96 [0.36-1.10]; P < 0.0001, Wilcoxon signed rank test). The median DNA concentration was not statistically different in samples amplified and not amplified, either for the WB4 (median 99 vs. 212 ng/μl; P = 0.120, Mann–Whitney test) or the WB20 (341 vs. 327 ng/μl; P = 0.780, Mann–Whitney test) dataset.

Coreceptor tropism inferred by the geno2pheno [coreceptor] algorithm was concordant in 18 of 20 randomly selected panel of 20 WB4 and WB20 paired samples, scoring 11 R5 and 7 non-R5 sequences (Table 3). Two samples yielded discordant results, one R5 in WB4 and non-R5 in WB20 (patient 8800) and one non-R5 in WB4 and R5 in WB20 (patient 5100). This was consistent with the discordance rate detected within duplicate analysis. Indeed, 2/20 of the WB4 (patients 196 and 5100) and 1/20 of the WB20 (patient 8800) duplicate sequences yielded one R5 and one non-R5 FPR value. The median ratio of highest to lowest FPR value within paired WB4 and WB20 duplicates was also comparable (median [IQR] 1.11 [0.9] for WB4 and 1.00 [0.54] for WB20; P = 0.538, Wilcoxon signed rank test).

**Table 3:** Geno2pheno [coreceptor] False Positive Rates (FPR) obtained from duplicate sequence analysis (designed A and B) of a randomly selected panel of 20 paired samples stored at +4°C (WB4) and at−20°C (WB20)

**Patient**	**WB4 FPR-A**	**WB4 FPR-B**	**WB4 tropism**	**WB20 FPR-A**	**WB20 FPR-B**	**WB20 tropism**
196	7.8	11.7	non-R5	7.9	6.0	non-R5
488	42.6	49.0	R5	49.0	49.0	R5
541	86.2	86.2	R5	87.4	86.2	R5
904	5.8	5.8	non-R5	5.8	5.8	non-R5
1339	10.8	10.8	R5	16.6	10.8	R5
1512	8.5	7.8	non-R5	8.5	8.5	non-R5
5012	7.8	4.8	non-R5	5.8	3.9	non-R5
5100	10.6	8.6	non-R5	12.0	12.0	R5
5656	83.0	83.0	R5	83.0	80.4	R5
6773	83.0	93.5	R5	48.4	48.4	R5
7055	1.7	1.7	non-R5	1.7	1.7	non-R5
8113	91.2	92.8	R5	92.8	92.8	R5
8800	17.1	17.1	R5	17.1	0.2	non-R5
9056	1.5	0.2	non-R5	0.1	1.6	non-R5
9746	17.8	46.7	R5	22.0	73.1	R5
9850	19.1	47.8	R5	47.8	47.8	R5
9880	94.1	95.2	R5	96.0	96.0	R5
10161	90.9	90.9	R5	90.9	90.9	R5
11659	0.1	0.5	non-R5	0.1	0.2	non-R5
11686	23.3	46.8	R5	35.3	23.3	R5

## Conclusions

This study shows that prolonged maintenance of whole blood at +4°C for two weeks and shipping at room temperature does not decrease the sensitivity of HIV-1 V3 DNA amplification compared with standard whole blood freezing followed by shipment in dry ice. The testing time schedule chosen fits well the clinical practice in most infectious diseases units sending samples to remote laboratories for genotypic coreceptor tropism.

Although prolonged whole blood storage at +4°C and shipment at room temperature results in some loss of DNA, this difference does not affect the V3 DNA amplification rate by the protocol used in this study. Higher purity of the DNA extracted from unfrozen vs. frozen blood, as suggested by the better OD_260/280_ ratio, likely explains this observation. Since DNA was not measured before PCR to make the procedure straightforward, it is currently uncertain whether and how much measurement and normalization of DNA input can improve sensitivity. Ultimately, the success of amplification is dependent on an optimal balance between the advantage of a larger DNA input and the possible drawbacks due to the parallel increase in PCR inhibitors derived from whole blood. Ideally, a thorough analysis of the interplay between these factors in individual samples could result in increased amplification rates. However, the 90% success rate obtained here makes the cost-effectiveness of individual sample analysis questionable, particularly in the context of suppressed HIV-1 viremia which can be associated with hardly detectable HIV-1 DNA independent from the method used [14].

In summary, storing whole blood at +4°C for up to two weeks and shipping at room temperature is a convenient method for obtaining HIV-1 gp120 V3 DNA sequence information via testing at a remote laboratory in patients with suppressed viremia. As expected, there is no preferential enrichment or loss of any virus sub-population, as detected by sequence variability and prediction of coreceptor tropism. In addition, this procedure can be adapted to amplification of any HIV-1 genome region and be useful also in resource-limited settings where HIV drug resistance surveillance requires simple, cost-effective and logistically feasible methods for sample collection and shipment to remote reference laboratories. As an additional pilot study (data not shown) we have obtained successful HIV-1 gp120 V3 DNA amplification from 11/11 whole blood samples stored at +4°C for two weeks and then placed at 37°C for two days to mimic shipment at the extreme temperatures typical of many resource-limited countries with high HIV prevalence. This preliminary evidence further supports straightforward working on HIV DNA without the need to implement cold chain management, resulting in expanded access to testing and cost savings.

## Abbreviations

HIV-1: Human immunodeficiency virus; RNA: Ribonucleic acid; DNA: Deoxyribonucleic acid; WB20: Whole blood stored at−20°C; WB4: Whole blood stored at + 4°C; PCR: Polymerase chain reaction; IQR: Interquartile range; OD260/280: Optical density ratio at 260 nm/280 nm; CD4: Cluster of differentiation 4; CCR5: C-C chemokine receptor type 5; CXCR4: C-X-C chemokine receptor type 4; V3: Third variable region of the HIV-1 gp120 envelope glycoprotein; ENV: Envelope; bp: Base pair.

## Competing interests

MZ has been a consultant to or has received research support or lecture fees from Abbott Pharmaceuticals, Abbott Molecular, Gilead Sciences, Janssen-Cilag, Merck Sharp and Dome, and ViiV Healthcare. AP and MM are employees of ViiV Healthcare. All authors declare that they have no competing interests.

## Authors’ contributions

MZ, AP, MM and GM designed the study. GM wrote the first draft. GM, AM, FS, IV, CC and AR performed the laboratory assays. All authors read and approved the final version of the manuscript.

## Pre-publication history

The pre-publication history for this paper can be accessed here:

http://www.biomedcentral.com/1471-2334/13/508/prepub
